# Progress of Surgery

**Published:** 1901-08-10

**Authors:** 


### PROGRESS OF SURGERY.

(*Continued from page* 284.)

### SURGERY OF THE BLADDER AND URETERS.

**Primary Tumours of the Renal Pelvis and Ureter.**—Albarran of Paris[5] has written important articles on these tumours and has collected forty-three cases, including those observed by himself. They may be divided into—I. The Epithelial Group, including (a) papillary tumours, and (b) sessile and infiltrating tumours; and II. The Connective Tissue Tumours. The papillary tumours are those most commonly met with. They nearly always arise in the pelvis, but may extend into the ureter and even along the ureter into the bladder. The growth in the ureter may be continuous with the original tumour, or appear as isolated tufts, probably of the nature of grafts from the parent growth. The villous tumours may be innocent or malignant. The latter are less finely villous and have a broader base of attachment usually, but in some cases the malignant invasion may be at one point only in the area of attachment to the pelvis. Albarran has no doubt that the innocent papilloma may become malignant. The malignancy may only show itself by recurrence after removal. The sessile tumours are cancers which may originate in the pelvis or ureter. They appear as nodular infiltrations and readily ulcerate. When the ureter is involved it becomes an indurated cord, with the lumen narrowed and irregular. The growths may be very malignant and spread to the kidney, lymphatic glands, peritoneum, liver and pleura. They frequently lead to hydro-, hæmo-, or pyo-nephrosis. It is significant that calculi were found in the pelvis in seven out of thirty-two cases. Clinically, like renal tumours, these epithelial growths produce the three cardinal symptoms of pain, bleeding, and the presence of a tumour in the loin. The treatment is by nephrectomy. The lumen of the ureter should be explored at the operation, and if there is evidence of its implication, it should be removed. If the vesical end of the ureter and adjacent wall of the bladder are involved, a second operation is required, viz., supra-pubic cystotomy to remove the stump of the ureter and the affected portion of the bladder. The connective tissue tumours of these parts are rare in comparison with the epithelial. It is almost impossible to differentiate them clinically from tumours of the kidney.

**Double Ureter.**—J. Edward Summers, jun., of Omaha, Nebraska,[6] has reported a case of nephrectomy upon a young child with this condition. The child was two and a half years old, and suffered from irritability of the bladder. The urine was acid, cloudy, and purulent, and contained tubercle bacilli in abundance. The left kidney was easily palpable. Before removing this organ the other kidney was palpated through a button-hole incision in the right loin. The affected left kidney had two ureters with separate pelves—an upper much enlarged tubercular tube, and a lower normal one. They were removed with the kidney, the former being followed well down to the pelvic brim. The patient made a good recovery. Summers is of opinion that these two ureters did not join before entering the bladder, otherwise they would have both shown tubercular disease.

**The Prevention of Urinary Stone Formation.**—Reginald Harrison[7] has contributed a paper on this subject. It appeared to this writer that the liability to recurrence of stone after lithotrity was larger than it should be and might be reduced, and that stone formation in all cases was more preventible by judicious treatment than it was generally considered to be. Cadge, in 1886, drew attention to the liability there was to recurrence of stone after lithotrity, and the liability is commonly held to somewhat discredit that operation. The liability, Harrison clearly shows, from his own experience of 23 cases of recurrence after 101 litholopaxies, is the greatest where the bladder has been functionally and structurally spoilt by the obstruction of an enlarged prostate. In the previously healthy, recurrence may of course occur from the descent of a fresh nucleus from the kidney, and this was liable to occur equally after lithotomy and after lithotrity. Harrison considers the preventive treatment under two headings: (1) the local treatment of the bladder following litholopaxy; (2) preventive treatment based upon the mode in which it is assumed that urinary calculi are formed, and finally he records some illustrative cases. He makes it a rule to regard all cases operated upon for stone as "suspicious" until repeated examinations of the urine show it to be normal. During this suspicious period the bladder should be washed out as for exploration. Often then a number of small phosphatic concretions or other crystalline bodies will be removed, which may be seen with a lense arranging themselves to form conglomerations of calculous material. To remove this material the bladder should be sluiced out rather than washed out in the ordinary sense. For this purpose a metal syringe which fits into the funnel-shaped end of a large-sized, smooth-bored, silk-web catheter, with an ample bevelled eye, should be used, and two to four pints of fluid—soft water, or solutions of sanitas, boric acid or permanganate of potash—injected daily. It often answers to fill the bladder fairly full and get the patient to lie or roll on the stomach. If the bladder is sacculated, and to make doubly sure, the evacuator catheter and aspirator, as for litholopaxy, may be used. Under this treatment the bladder becomes gradually less liable to form these concretions. If the mucous membrane of the bladder remains lax and spongy, as evidenced by an excess of mucus in the urine, it is well, after the sluicing, to inject and leave in it a couple of ounces of a solution of nitrate of silver (one grain to twelve ounces) two or three times a week. Harrison then discusses briefly the mode of formation of urinary calculi by what Rainey termed "molecular coalescence," and points out that a multitude of factors or conditions are necessary for the formation of stone, and that hitherto, in treatment, attention has been too exclusively paid to the factor arising from the presence in the urine of excess of the characteristic ingredients of stones. Other contributory causes capable of bringing about consolidation of that which is natural in the excretion have been neglected. Excess of uric acid in the urine, for instance, does not seem to be the cause of the frequency of stone in the East Indian, but rather the power he possesses of converting into stone all the uric acid that he gets from a diet which is chiefly non-nitrogenous. It is one thing to prevent a stone forming and another to diminish the size of a stone already existing, but remedies such as those containing alkalies may be useful for both purposes. Probably the spas of Contrexéville, Wildungen, Vichy and others are useful preventives of stone. Harrison has observed that drinking hard water has sometimes caused an increase in the amount of mucus in the urine, especially in children, and this may favour molecular coalescence. Cadge observed that children in the Eastern Counties of England, where stone is prevalent, were often reared without much milk being given them. Harrison has found also that persons, who have been in the habit of passing small urate and oxalate calculi have sometimes apparently ceased to do so after taking from time to time short courses of such drugs as turpentine, sandal and copaiba. An uncleanly condition of the urine, particularly residual urine, is often a contributory cause of ammoniacal decomposition and the recurrence of stone after operation. Phosphatic calculi may form without any other visible nuclei other than the concretion of the crystals of triple phosphate. To cleanse the urine, besides the sluicing described above, such drugs as sandal wood, cubebs, turpentine, and, better, a digestible and easily soluble salt, the borocitrate of magnesia, were useful. The latter is taken in teaspoonful doses in a tumbler of water. Many people use it as freely as common salt with their food. Other useful drugs are salicylate of sodium, benzoate of sodium, benzoate of ammonium, hyposulphite of sodium, and urotropine.

**Lateral Lithotomy.**—T. Vincent Jackson[8] has published, with comments, the record of a successful case of lateral lithotomy. The patient, a boy of seven, had had painful micturition for three years. There was pus in the urine, but no blood or casts. The stone was first seized in the points of a lithotrite. The blades of the instrument had to be separated fully two inches to grasp the stone. This showed that the stone was a hard one and of unusual bulk. It was decided, therefore, to perform lithotomy rather than lithotrity. The latter was easily accomplished in two minutes. Rapid convalescence followed, the boy getting up in a fortnight, and leaving the hospital in 23 days. The stone weighed 2 ounces and 2 drachms. Jackson remarks that crushing rather than cutting is the operation which should be selected for stone unless the size of the stone or the local conditions are unfavourable. A case of R. Caldecott's (*Indian Med. Gazette*, Dec. 1886) is alluded to, in which a patient died shortly after a crushing operation, the cause of death being peritonitis induced by a sharp fragment of stone having pierced the wall of the bladder and lying in the rent. Death followed two days after lithotrity in another case, the operation having lasted 71 minutes. Lithotomy, Vincent remarks, might have saved these lives. The dictum has been enunciated that every stone weighing two ounces and over is to be regarded as a large stone and dealt with accordingly. The rapid and easy recovery of Vincent's patient justified his selection of the operation of lateral lithotomy.

[5] *Vide* Abst. Edin. Med. Jour., 1901. [6] Anns. of Surg., Jan., 1901. [7] The Lancet, Feb., 1901. [8] The Lancet, March, 1901.

